# Perceived stigma, self stigma and barriers to treatment in alcohol dependent individuals

**DOI:** 10.1192/j.eurpsy.2023.1404

**Published:** 2023-07-19

**Authors:** R. Tripathi, S. Singh, M. Prithviraj, A. Nair

**Affiliations:** All India Institute of Medical Sciences, Gorakhpur, India

## Abstract

**Introduction:**

Alcohol use disorder is a chronic relapsing disorder. It is a matter of global health concern affecting different countries, cultures, economic classes and ethnic groups. Although, many people benefit from alcohol related treatment, low occurrence of treatment seeking is a common denominator for the majority of people suffering from alcohol use disorder.

**Objectives:**

The aim of the present study was to study barriers of treatment seeking and assess self and perceived stigma in alcohol dependent male patients in rural population of India

**Methods:**

An observational study was conducted at a private de-addiction center in India. Male patients who were more than 18 years old, alcohol dependent with more than seven days of admission (not currently in withdrawal) were included in the study

**Results:**

The mean age of the sample was 29.1 (7.8) years and age of onset of alcohol use was 18.5 (3.3) years. The mean quantity of alcohol used per day was around 550 millilitres of IMFL per day. The mean number of previous abstinent attempt were two. The most common barrier to treatment was financial (poor affordability). Not serious enough to change and being afraid of what others might think (stigma) were other common barriers. The mean value of perceived stigma was 21.9 (2.3). No co-relation was observed between stigma (both perceived and self stigma) and age of onset and quantity of alcohol consumed

**Conclusions:**

These barriers and stigma needs to be addressed to improve treatment seeking and reduce relapse in our population

**Disclosure of Interest:**

None Declared

